# Adverse Event Landscape of Pulsed Field Ablation Catheters: A Computational Review of FDA MAUDE Post-Market Surveillance Data

**DOI:** 10.31083/RCM53385

**Published:** 2026-09-11

**Authors:** Matthew W. Segar, Kaleb D. Lambeth, Shyon Parsa, Mehdi Razavi

**Affiliations:** ^1^Department of Cardiology, Texas Heart Institute, Houston, TX 77030, USA; ^2^Department of Medicine, Stanford University School of Medicine, Stanford, CA 94305, USA; ^3^Department of Cardiology, Baylor College of Medicine, Houston, TX 77030, USA

**Keywords:** catheter ablation, atrial fibrillation, natural language processing, postmarketing product surveillance, equipment failure, United States Food and Drug Administration

## Abstract

**Background:**

Pulsed field ablation (PFA) is Food and Drug Administration (FDA)-cleared for pulmonary vein isolation in atrial fibrillation. Using a keyword-based natural language processing pipeline applied to free-text narratives in the FDA MAUDE database, we aimed to characterize and compare adverse event and device malfunction profiles across the four cleared PFA systems.

**Methods:**

We retrieved 6930 MAUDE reports for FDA product code QZI (pulsed field ablation catheter) spanning December 13, 2023 through April 30, 2026, covering all four FDA-cleared PFA systems. Reports were deduplicated and assigned to a specific device system using manufacturer and brand name fields. Concatenated free-text narratives were classified into 11 predefined adverse event categories using multi-label keyword-based natural language processing. Device malfunction reports were additionally subclassified into one of 11 hardware-specific subtypes. Descriptive statistics were used throughout.

**Results:**

Of 6930 reports, 4185 (60.4%) were classified into at least one adverse event category. The three most common categories were device/catheter malfunction (2068; 29.8%), cardiac tamponade/pericardial effusion (915; 13.2%), and stroke/TIA/cerebral embolism (600; 8.7%). PFA-specific physiologic adverse events—ST-elevation myocardial infarction (STEMI), hemolysis/acute kidney injury, and phrenic nerve palsy—accounted for 615 combined reports (8.9% of all reports). Fatality proportions were highest for vascular access complications (6.8%), esophageal/atrioesophageal injury (6.0%), and coronary artery spasm/STEMI (5.3%). Among malfunction reports, retraction/retrieval failure predominated for Farapulse (48.9%), while electrical/energy delivery failure was the leading subtype for PulseSelect (29.4%).

**Conclusions:**

Natural language processing (NLP)-assisted keyword classification enables systematic, scalable characterization of adverse event patterns across nearly 7000 real-world PFA reports. PFA-specific physiologic adverse events are identifiable through post-market surveillance data and constitute a distinct safety phenotype separate from device malfunction. This approach provides a reproducible framework for near-real-time surveillance of novel cardiac ablation systems.

## 1. Introduction

Pulsed field ablation (PFA) delivers ultrashort, high-voltage electrical pulses that cause irreversible electroporation of cardiomyocytes through a non-thermal mechanism. The biophysical selectivity of this approach for cardiac tissue has driven rapid clinical adoption of PFA for pulmonary vein isolation (PVI) in atrial fibrillation, with demonstrated reductions in atrioesophageal fistula, pulmonary vein stenosis, and phrenic nerve injury compared with radiofrequency and cryoablation [1,2,3]. The Food and Drug Administration (FDA) cleared four PFA systems for PVI between December 2023 and November 2024: PulseSelect (Medtronic; Dec 13, 2023), Farapulse (Boston Scientific; Jan 30, 2024), Sphere-9/Affera (Medtronic; Oct 24, 2024), and Varipulse (Biosense Webster/J&J; Nov 6, 2024) [4,5,6,7]. Each system embodies a distinct catheter architecture and pulse delivery strategy, which may translate into different real-world safety profiles.

While pivotal trials and early registries have established the broad safety of PFA, they have also identified physiologic adverse events specific to electroporation. Coronary artery spasm arises from electroporation of perivascular autonomic ganglia and vascular smooth muscle, particularly during cavotricuspid or mitral isthmus ablation near the right or left circumflex coronary artery [8,9,10]. Intravascular hemolysis results from direct erythrocyte lysis in high-energy electric fields and can cause hemoglobinuria-mediated acute kidney injury (AKI) in a dose-dependent fashion [11,12]. Phrenic nerve palsy, while less frequent with PFA than with cryoablation, continues to be reported across systems [13].

Post-market surveillance through the Manufacturer and User Facility Device Experience (MAUDE) database represents the most comprehensive source of real-world adverse event data for FDA-cleared devices, but free-text narratives render large-scale manual review impractical. A prior published MAUDE analysis covering two PFA systems during the first seven months after clearance identified 156 clinical adverse event reports [14]. That analysis was limited to Farapulse and PulseSelect and predated the clearance of Sphere-9/Affera and Varipulse. This study characterizes the complete adverse event landscape across 6930 MAUDE reports for all four FDA-cleared PFA systems using a reproducible, keyword-based natural language processing (NLP) pipeline, with secondary objectives of comparing adverse event profiles across device systems and characterizing malfunction subtypes by catheter architecture.

## 2. Methods

### 2.1 Data Source and Device System Classification

MAUDE adverse event reports were retrieved from the openFDA device adverse event application programming interface (API) using product code QZI (pulsed field ablation catheter) [15]. The study period spanned December 13, 2023, the date of the first PFA system clearance, through April 30, 2026. Duplicate reports were removed prior to analysis. The following fields were extracted for each report: brand name, manufacturer name, event type, patient outcome, date received, event description, and additional narrative.

Each report was assigned to one of four named PFA systems or an unclassified category using brand name and manufacturer name fields. Brand name was used as the primary identifier, with manufacturer name as a fallback for reports where the brand name was absent or non-specific. Sphere-9/Affera was classified before PulseSelect to resolve ambiguity between systems sharing the same manufacturer. Reports that could not be assigned to a specific named system were excluded from device-level comparisons but retained in pooled analyses.

### 2.2 Narrative Construction

The analytical narrative for each report was constructed by concatenating the event description and additional narrative fields. This combined text served as the input to all NLP classification steps. Reports with no analyzable narrative text were retained in report-level counts but excluded from NLP classification denominators.

### 2.3 Adverse Event and Device Malfunction Classifications

Eleven adverse event categories were defined a priori based on adverse events documented in the published PFA clinical literature. Categories and their representative keyword terms are provided in **Supplementary Table 1**. Classification used case-insensitive keyword matching applied independently to each category. The pipeline was multi-label: a single report could match any number of categories simultaneously.

Reports matching the device/catheter malfunction category were additionally classified into one of 11 hardware-specific subtypes using a separate first-match keyword hierarchy. Unlike the multi-label adverse event pipeline, each malfunction report received exactly one subtype, the first whose pattern matched the narrative; reports with no match were assigned to Other/Unspecified. The order was fixed to resolve pattern overlap rather than to rank clinical severity, with more specific failure modes evaluated before broader ones (e.g., electrical delivery failure before mechanical damage) so a narrative describing a specific failure was not captured by a more general pattern. Patterns were designed to reflect the known architectural characteristics of each catheter platform.

### 2.4 Outcome Extraction

Event type (Death, Injury, Malfunction) was extracted from the MAUDE event type structured field. Patient outcomes were derived from the patient outcome field; a report was flagged as life-threatening, associated with hospitalization, or requiring intervention based on the reporter-assigned designations in that field. Patient outcomes are not mutually exclusive in MAUDE reporting and represent the mandatory reporter’s characterization at time of submission.

### 2.5 Statistical Analysis

All analyses were descriptive. Proportions are reported as percentages of the relevant denominator. Monthly report volume was calculated by assigning each report to a calendar month based on the date received. Device approval dates were overlaid as reference markers. Cumulative report counts were stratified by device system and plotted as a stacked bar chart to characterize the post-market adoption trajectory for each system. Device-level adverse event comparisons were restricted to device systems with at least 200 reports. All four named systems met this threshold. Proportions reflect each device system’s own report total as the denominator and do not represent per-procedure event rates, as MAUDE does not capture procedure volume; no inferential comparisons between systems were performed. No inferential comparisons between device systems were performed, given differences in time on market, cumulative report volume, and manufacturer reporting practices. All analyses were conducted in Python 3.11 using *pandas* (pandas version 2.3.3, NumFOCUS, Austin, TX, USA) and *matplotlib *(matplotlib version 3.10.0, NumFOCUS, Austin, TX, USA). Study flow was rendered using Graphviz.

## 3. Results

A total of 6930 unique MAUDE reports were identified for product code QZI across the study period (Fig. 1). Of these, 6915 (99.8%) were attributable to one of the four named PFA systems (Table 1). Farapulse accounted for 4373 reports (63.1%), PulseSelect contributed 1569 (22.6%), Sphere-9/Affera 570 (8.2%), and Varipulse 403 (5.8%). Fifteen reports could not be assigned to a specific system. Trends in monthly MAUDE reports by catheter are shown in Fig. 2.

**Fig. 1.**
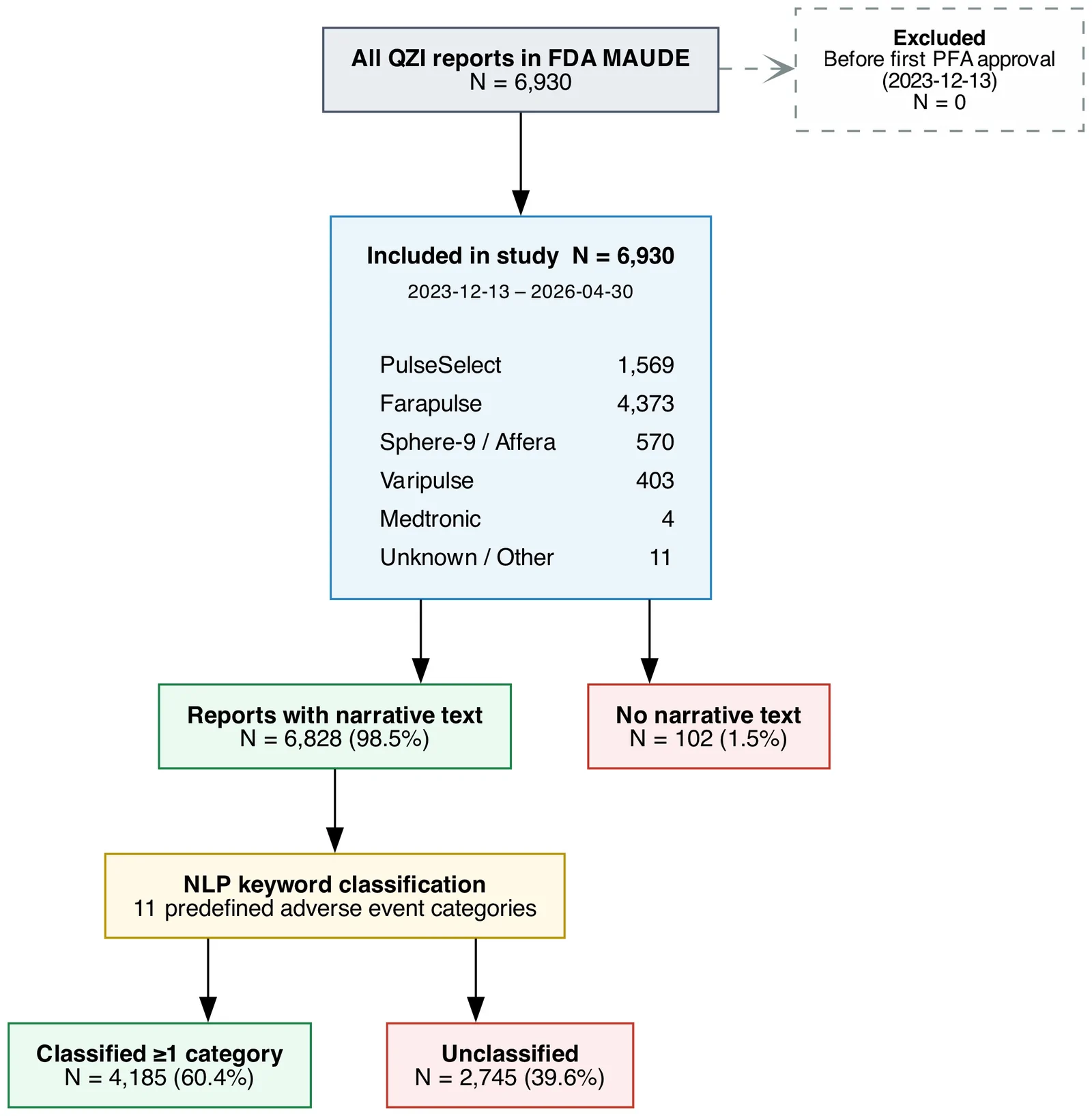
**Study flow diagram**. FDA, Food and Drug Administration; MAUDE, Manufacturer and User Facility Device Experience; NLP, natural language processing.

**Fig. 2.**
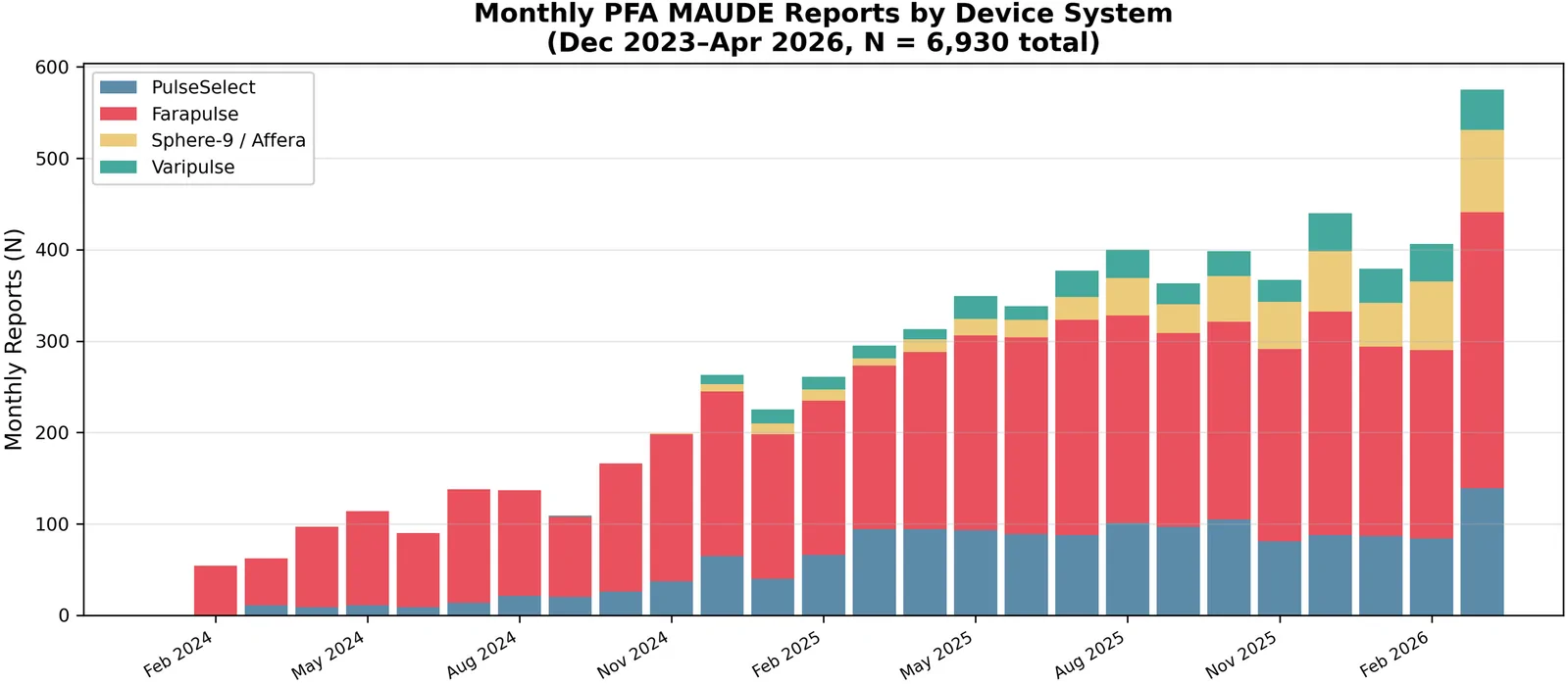
**Monthly PFA MAUDE reports by device system (December 2023–April 2026)**.

**Table 1.** **Characteristics of FDA MAUDE reports by PFA device system**.

Device System	FDA Clearance	Reports, n	Death, n (%)	Injury, n (%)	Malfunction, n (%)	Life-Threatening, n (%)	Hospitalization, n (%)
PulseSelect (Medtronic)	Dec 13, 2023	1569	15 (1.0)	457 (29.1)	1097 (69.9)	60 (3.8)	89 (5.7)
Farapulse (Boston Scientific)	Jan 30, 2024	4373	140 (3.2)	1790 (40.9)	2440 (55.8)	28 (0.6)	376 (8.6)
Sphere-9/Affera (Medtronic)	Oct 24, 2024	570	14 (2.5)	388 (68.1)	168 (29.5)	68 (11.9)	110 (19.3)
Varipulse (Biosense Webster/J&J)	Nov 6, 2024	403	4 (1.0)	289 (71.7)	110 (27.3)	124 (30.8)	68 (16.9)
Total (named systems)		6915	173 (2.5)	2924 (42.3)	3815 (55.2)	280 (4.0)	643 (9.3)

Event type and outcome percentages are calculated as a proportion of total reports for each device system. Life-threatening and hospitalization are derived from the MAUDE *patient_outcome* field and are not mutually exclusive. Total row reflects the four named systems (N = 6915); 15 additional reports from unidentified systems are excluded. PFA, pulsed field ablation; MAUDE, Manufacturer and User Facility Device Experience; FDA, Food and Drug Administration.

Across all reports, malfunction was the most common event type (3815; 55.2%), followed by injury (2924; 42.3%) and death (173; 2.5%). Among outcome designations, 4.0% of reports were classified as life-threatening, 9.3% resulted in hospitalization, and 18.2% required intervention. Analyzable narrative text was present in 98.5% of reports (6828 of 6930).

### 3.1 Adverse Event Categories

Of 6930 reports, 4185 (60.4%) were classified into at least one adverse event category. The remaining 2745 (39.6%) were unclassified. Among classified reports, the mean number of categories per report was 1.26 [SD 0.82; range 1–8]. Results are summarized in Table 2 and Fig.3. Device/catheter malfunction was the most frequent category (2068 reports; 29.8%). Cardiac tamponade/pericardial effusion ranked second (915; 13.2%), with 4.9% of these associated with a death event type and 7.1% designated life-threatening. Stroke, transient ischemic attack (TIA), or cerebral embolism accounted for 600 reports (8.7%) and carried the highest life-threatening designation rate of any category (16.8%).

**Table 2.** **Adverse event categories by NLP keyword classification (pooled, N = 6930 reports)**.

Rank	Adverse Event Category	Reports, n (% total)	Death, n (%)	Injury, n (%)	Malfunction, n (%)	Life-Threatening, n (%)
1	Device/catheter malfunction	2068 (29.8)	11 (0.5)	226 (10.9)	1831 (88.5)	31 (1.5)
2	Cardiac tamponade/pericardial effusion	915 (13.2)	45 (4.9)	864 (94.4)	5 (0.5)	65 (7.1)
3	Stroke/TIA/cerebral embolism	600 (8.7)	18 (3.0)	559 (93.2)	23 (3.8)	101 (16.8)
4	Heart block/conduction disturbance	408 (5.9)	18 (4.4)	383 (93.9)	7 (1.7)	39 (9.6)
5	Atrial flutter/arrhythmia recurrence	326 (4.7)	14 (4.3)	272 (83.4)	40 (12.3)	18 (5.5)
6	Coronary artery spasm/STEMI	319 (4.6)	17 (5.3)	297 (93.1)	5 (1.6)	20 (6.3)
7	Esophageal/atrioesophageal injury	215 (3.1)	13 (6.0)	196 (91.2)	6 (2.8)	22 (10.2)
8	Hemolysis/acute kidney injury	166 (2.4)	7 (4.2)	159 (95.8)	0 (0.0)	13 (7.8)
9	Phrenic nerve palsy	130 (1.9)	1 (0.8)	128 (98.5)	1 (0.8)	1 (0.8)
10	Vascular access complications	103 (1.5)	7 (6.8)	95 (92.2)	1 (1.0)	7 (6.8)
11	Pulmonary vein stenosis	28 (0.4)	0 (0.0)	21 (75.0)	7 (25.0)	1 (3.6)
—	Any category (classified)	4185 (60.4)				
—	Unclassified	2745 (39.6)				

Categories are not mutually exclusive; a single report may match more than one category. Death, Injury, and Malfunction percentages reflect the proportion within each category assigned that event type. Life-threatening is derived from the MAUDE *patient_outcome* field. NLP, natural language processing; TIA, transient ischemic attack; STEMI, ST-elevation myocardial infarction.

**Fig. 3.**
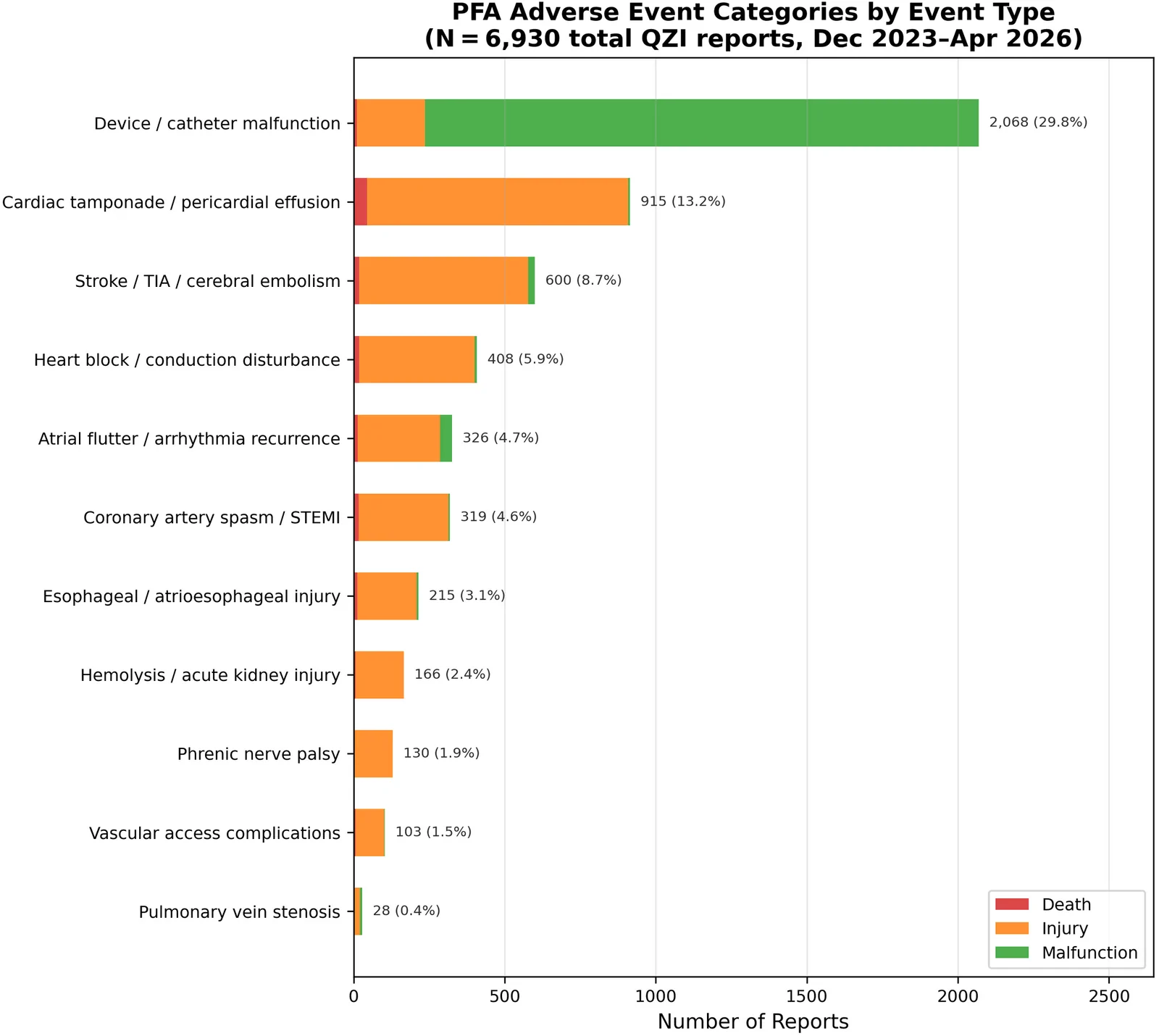
**PFA adverse event categories by event type (N = 6930 QZI reports, December 2023–April 2026)**. TIA, transient ischemic attack; STEMI, ST-elevation myocardial infarction.

Fatality proportions were highest for vascular access complications (6.8%), esophageal/atrioesophageal injury (6.0%), and ST-elevation myocardial infarction (STEMI) (5.3%). Esophageal injury represented only 3.1% of total reports but carried a 10.2% life-threatening rate, consistent with the severity of atrioesophageal fistula as a procedure-related complication.

PFA-specific physiologic adverse events [coronary artery spasm/STEMI (319 reports; 4.6%), hemolysis/AKI (166; 2.4%), and phrenic nerve palsy (130; 1.9%)] collectively accounted for 615 reports (8.9% of all reports; 14.7% of classified reports). Phrenic nerve palsy had the lowest fatality proportion (0.8%) and life-threatening rate (0.8%) of any clinical adverse event category, consistent with its typically self-limited course.

### 3.2 Adverse Event Profiles by Device System

Adverse event distributions varied across device systems (Table 3). Device/catheter malfunction was most common for PulseSelect (37.0%) and least common for Sphere-9/Affera (9.3%). Cardiac tamponade/pericardial effusion was most frequent for Farapulse (15.6%) and Varipulse (17.9%). Varipulse had the highest proportion of stroke/TIA reports (40.2%), more than four times the reporting of any other system. Sphere-9/Affera had a disproportionately high reporting of heart block/conduction disturbance (14.4%), however this may reflect its use in ventricular and complex atrial substrates beyond PVI.

**Table 3.** **Adverse event categories by device system**.

Adverse Event Category	PulseSelect (n = 1569)	Farapulse (n = 4373)	Sphere-9/Affera (n = 570)	Varipulse (n = 403)
Device/catheter malfunction	581 (37.0)	1343 (30.7)	53 (9.3)	88 (21.8)
Cardiac tamponade/pericardial effusion	108 (6.9)	684 (15.6)	47 (8.2)	72 (17.9)
Stroke/TIA/cerebral embolism	83 (5.3)	301 (6.9)	54 (9.5)	162 (40.2)
Heart block/conduction disturbance	78 (5.0)	229 (5.2)	82 (14.4)	19 (4.7)
Atrial flutter/arrhythmia recurrence	22 (1.4)	247 (5.6)	30 (5.3)	27 (6.7)
Coronary artery spasm/STEMI	48 (3.1)	230 (5.3)	27 (4.7)	13 (3.2)
Esophageal/atrioesophageal injury	10 (0.6)	158 (3.6)	9 (1.6)	38 (9.4)
Hemolysis/acute kidney injury	8 (0.5)	132 (3.0)	11 (1.9)	15 (3.7)

Data presented as n (% of total reports for that device system). Only device systems with N ≥200 reports are shown; all four named systems met this threshold. Categories are not mutually exclusive. Approval dates differ across systems (range: December 2023 to November 2024); report volume reflects cumulative time on market and does not represent per-procedure event rates. TIA, transient ischemic attack; STEMI, ST-elevation myocardial infarction.

### 3.3 Device Malfunction Subtypes

Among 2068 malfunction reports, subtype distributions differed substantially by device system (Fig. 4 and **Supplementary Table 2**). Retraction/retrieval failure was the dominant pooled subtype (802; 38.8%) and was most pronounced for Farapulse (48.9% of its malfunction reports), consistent with the collapsible pentaspline basket design requiring reliable re-engagement with the delivery sheath after each deployment. Electrical/energy delivery failure accounted for the largest proportion of malfunction reports for PulseSelect (29.4%) and Varipulse (20.5%), reflecting generator-level alarm events and energy delivery interruptions characteristic of those systems. Sphere-9/Affera malfunction reports were most commonly unclassified (32.1%), with disproportionate software/sensor failure (13.2%) and contamination/sterility breach (5.7%), likely reflecting its multimodal radiofrequency (RF)-plus-PFA design and failure modes not captured by the primary PFA basket catheter taxonomy.

**Fig. 4.**
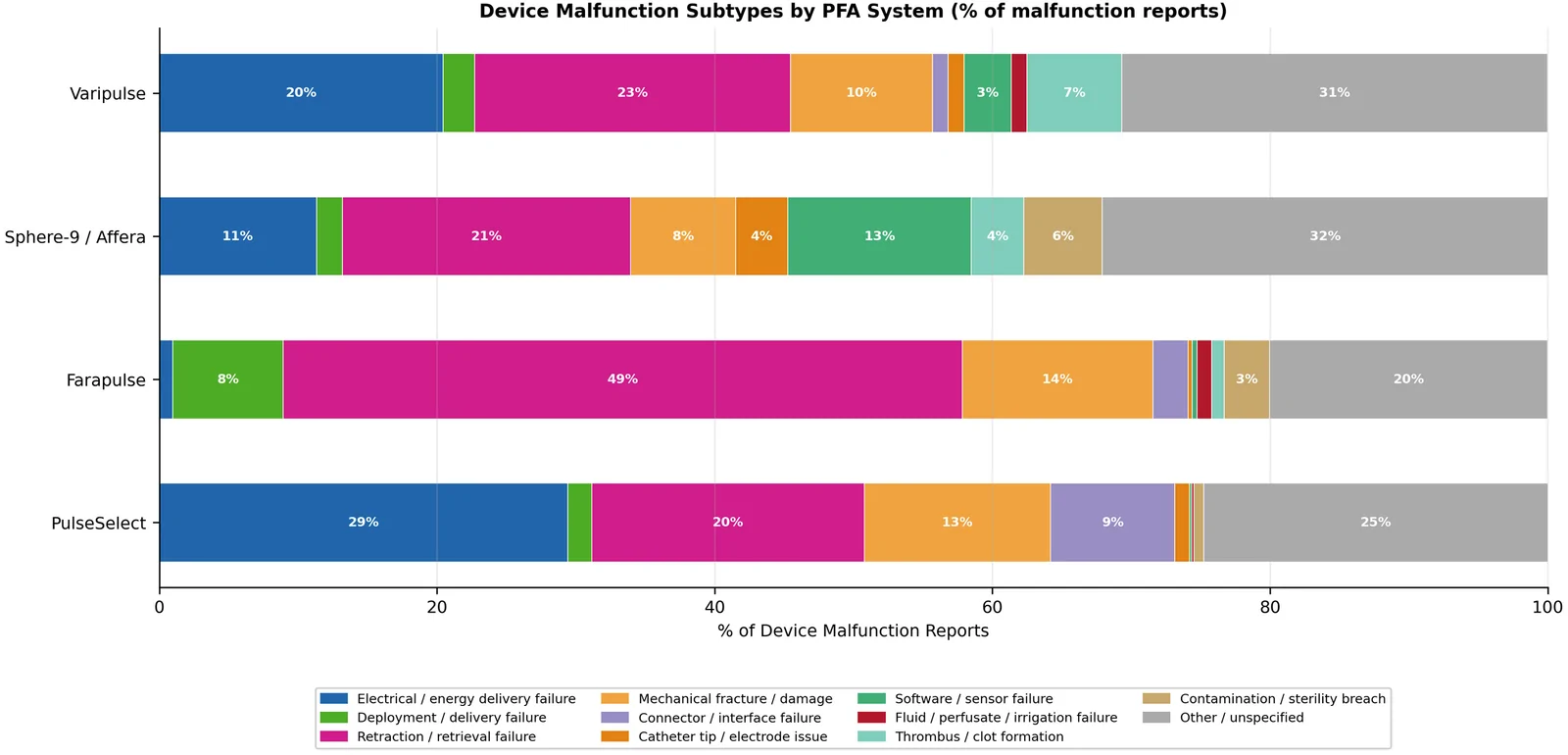
**Device malfunction subtypes by PFA system (% of device malfunction reports)**.

## 4. Discussion

In this computational analysis of 6930 FDA MAUDE reports spanning the complete post-market experience of all four FDA-cleared PFA systems, we observed several key findings. First, device malfunction was the dominant adverse event category, accounting for nearly 30% of all reports and comprising a distinct pattern of catheter-architecture-specific failure modes. Second, serious clinical adverse events (cardiac tamponade, stroke, and heart block) occurred across all systems and collectively carried substantial mortality and morbidity burden within the reporting dataset. Third, PFA-specific physiologic adverse events coronary artery spasm, hemolysis with acute kidney injury, and phrenic nerve palsy arise from mechanisms specific to electroporation rather than from thermal injury, distinguishing them from complications shared across ablation modalities. Direct MAUDE comparison supports this distinction: in the prior PFA-versus-radiofrequency analysis, hemolysis was reported in 9.0% of PFA adverse events versus none for radiofrequency [16]. Fourth, adverse event profiles differed meaningfully across device systems in patterns that correspond to known differences in catheter architecture and pulse delivery design.

Device malfunction constituted the most frequent category overall and was the dominant event type for PulseSelect (37.0%) and a substantial proportion for Farapulse (30.7%). This pattern is consistent with the early post-market period for novel cardiac catheters, during which operator unfamiliarity with catheter handling, sheath re-engagement, and generator behavior commonly generates malfunction reports. In the prior MAUDE comparison of PFA versus radiofrequency ablation across January through July 2024, Cho et al. [16] similarly found that mechanical problems represented 87.2% of PFA malfunction reports, compared with 17.2% for radiofrequency catheters, highlighting the degree to which early PFA adoption was dominated by hardware-related reporting. Malfunction subtype analysis in the present study revealed architecture-specific patterns: retraction and retrieval failure predominated for Farapulse (48.9%), reflecting the collapsible pentaspline basket design that requires reliable sheath re-engagement after each deployment, while electrical and energy delivery failures were more prominent for PulseSelect (29.4%) and Varipulse (20.5%), consistent with generator-level alarm behavior in those systems. These findings have direct implications for operator training. Programs introducing a new PFA system should anticipate a learning curve weighted toward catheter handling and generator management, and structured proctoring programs should address these failure modes explicitly.

Cardiac tamponade was the second most common category (13.2% of all reports), with a 4.9% fatality proportion and 7.1% life-threatening rate. These proportions reflect mandatory reporting of clinically significant events rather than overall procedural risk. For context, the MANIFEST-17K registry, which prospectively captured post-approval Farapulse procedures across 106 centers and 17,642 patients, reported pericardial tamponade in 0.36% of procedures [13]. The substantially higher proportion in MAUDE reflects the well-characterized denominator problem: MAUDE captures adverse events without a denominator of procedures performed, selectively enriching for more severe or reportable events. Stroke and cerebral embolism (8.7% of reports) carried the highest life-threatening designation rate of any clinical category (16.8%), representing the most consequential neurological risk of the procedure.

Coronary artery spasm and STEMI accounted for 319 reports (4.6%) and carried a 5.3% fatality proportion. This complication arises from electroporation of perivascular autonomic ganglia and direct vascular smooth muscle activation, particularly during ablation near the cavotricuspid or mitral isthmus [9,10]. The NEMESIS-PFA registry, which compared biomarker profiles across 871 patients undergoing PFA versus radiofrequency ablation, demonstrated significantly greater increases in cardiac troponin, lactate dehydrogenase (LDH), and other markers of myocardial stress with PFA, suggesting that energy-related collateral effects on adjacent structures may be more widespread than captured by clinical event counts alone [17]. Preclinical data have raised concern for longer-term coronary arterial injury, including intimal hyperplasia and tunica media fibrosis, at ablation sites adjacent to prior vasospasm [18]. Hemolysis and AKI accounted for 166 reports (2.4%). Intravascular hemolysis results from direct erythrocyte membrane disruption in high-energy electric fields, and its clinical severity is dose-dependent on application number and catheter-tissue contact [11,12,19]. In the Cho et al. [16] MAUDE comparison, hemolysis was reported in 9.0% of PFA adverse events versus 0% for radiofrequency ablation, confirming that this is an energy-specific rather than a procedural-class complication. Phrenic nerve palsy (130 reports; 1.9%) carried the lowest fatality proportion (0.8%) and life-threatening rate (0.8%) of any clinical category, consistent with its predominantly transient course. Registry data confirm that persistent phrenic palsy is rare with PFA. In MANIFEST-17K, transient phrenic palsy occurred in 0.06% of patients and no persistent cases were reported [13]. Meta-analytic data estimate the phrenic nerve injury rate at 0.23% with PFA compared with 2.68% with cryoballoon ablation representing one of the clearest safety advantages of PFA over cryoenergy [20].

The disproportionately high stroke/TIA proportion for Varipulse (40.2%) merits dedicated discussion. This finding is based on 403 reports over a short post-market window following November 2024 clearance and must be interpreted in the context of publicly available safety communications. In early 2025, the manufacturer issued a field safety notice after four neurovascular events, and a temporary clinical hold was implemented pending investigation. The manufacturer attributed increased risk to high application counts, stacking of applications, and ablation outside the pulmonary veins. This MAUDE signal is therefore consistent with the reported mechanism and underscores the value of near-real-time surveillance. The elevated stroke proportion was detectable in the aggregate reporting data concurrent with, and potentially before, the formal safety communication. However, the elevated stroke/TIA proportion for Varipulse should not be read as a per-procedure risk estimate. It reflects a small report volume over a short window following November 2024 clearance and is most appropriately interpreted alongside the manufacturer’s 2025 field safety communication.

This study is approximately sevenfold larger than the prior published MAUDE analysis of PFA, which examined 156 PFA clinical adverse event reports from January through July 2024 and was limited to two device systems [14]. Extending the analysis to all four cleared systems and a longer surveillance window substantially improves the precision of rare event estimates. Importantly, MAUDE and prospective registries such as MANIFEST-17K and MANIFEST-PF serve complementary rather than competing roles [13,21]. Registries provide denominator-adjusted event rates and standardized outcome definitions. MAUDE, conversely, provides rapid, population-level signal detection across all centers and indications, including applications outside study-defined protocols. NLP-assisted classification enables MAUDE to function as an early warning system for emerging signals (such as the Varipulse stroke pattern) before formal registry data mature.

These findings have several implications for clinical practice and program development. The malfunction subtype profiles suggest that structured catheter-specific training, particularly for sheath re-engagement with basket catheters and generator alarm management, should be incorporated into credentialing programs for each PFA system. The prevalence of coronary artery spasm reports supports routine availability of intracoronary or intravenous nitroglycerin for any procedure involving isthmus ablation with PFA. The hemolysis signal supports adoption of application-count awareness and post-procedural hydration protocols in programs performing high-lesion-burden PFA. The esophageal injury category (215 reports; 3.1% with 6.0% fatality proportion) is a notable finding given that esophageal protection was a primary motivation for PFA adoption; these reports may reflect thermal contributions from Sphere-9/Affera’s radiofrequency capability, cases with atypical esophageal anatomy, or procedures involving posterior wall ablation, and should prompt continued vigilance.

### Limitations

Several limitations must be acknowledged. First, MAUDE is a passive, voluntary reporting system subject to substantial under-reporting; the true incidence of all adverse events exceeds what is captured here. Second, MAUDE does not record procedure volume, so all proportions reflect the distribution of reported events rather than per-procedure incidence and cannot be interpreted as comparative risk between systems. Third, keyword classification was not validated against a manually adjudicated gold standard, and the absence of negation detection may produce false-positive assignments when an adverse event is explicitly denied in the narrative. Fourth, device-level comparisons are confounded by differences in time on market, cumulative procedure volume, and manufacturer reporting practices. Fifth, the elevated stroke/TIA proportion for Varipulse reflects a small sample (n = 403) and a short post-market window and should be interpreted alongside the manufacturer’s 2025 safety communication. Sixth, since the Sphere-9/Affera system delivers both RF and PFA, its adverse event and malfunction profiles likely reflect a mix of energy-source contributions that the present classification does not distinguish.

## 5. Conclusions

NLP-assisted keyword classification of 6930 FDA MAUDE reports characterizes the real-world adverse event landscape of pulsed field ablation catheters across all four FDA-cleared systems. Device malfunction predominates during the early post-market period, and its subtype distribution reflects catheter architecture differences across platforms. PFA-specific physiologic adverse events (coronary artery spasm, hemolysis/AKI, and phrenic nerve palsy) are identifiable in post-market surveillance data and constitute a distinct safety phenotype separate from procedural complications shared with thermal ablation. Stroke and esophageal injury carry the highest severity burden within the clinical categories. The computational approach described here is scalable, reproducible, and updatable in near-real time, providing a practical framework for ongoing post-market surveillance of novel cardiac ablation systems.

## Data Availability

MAUDE data are publicly available via the openFDA API (https://open.fda.gov/apis/device/event/). Analysis code is available from the corresponding author upon reasonable request.
