# Supplementary material for: Adverse Event Landscape of Pulsed Field Ablation Catheters: A Computational Review of FDA MAUDE Post-Market Surveillance Data
Source: Rev Cardiovasc Med. 2026 Sep 11;27(9):53385. doi: 10.31083/RCM53385 (PMC13653163; doi:10.31083/RCM53385)
Supplement: Supplementary file 1 [file 2153-8174-27-9-53385-s1.zip › Supplementary Material.docx]

## **Supplementary Material**

**Supplementary Table 1.** Adverse Event Classification Categories and Representative Keyword Terms

| **Adverse Event Category** | **Representative Keyword Terms** |
| --- | --- |
| Device/catheter malfunction | malfunction, device failure, catheter failure, retract, stuck, dislodge, defect, damaged, fracture, disconnect |
| Coronary artery spasm/STEMI | coronary spasm, vasospasm, STEMI, ST-elevation, troponin, coronary angiograph |
| Cardiac tamponade/pericardial effusion | tamponade, pericardial effusion, pericardiocentesis, hemopericardium, pericarditis |
| Hemolysis/acute kidney injury | hemolysis, haemolysis, hemoglobinuria, acute kidney, AKI, creatinine elevated, dialysis |
| Stroke/TIA/cerebral embolism | stroke, transient ischemic, TIA, cerebral embolism, cerebral infarct, aphasia, hemiplegia, air embolism, thromboemboli |
| Heart block/conduction disturbance | heart block, AV block, complete heart block, bradycardia, asystole, pacemaker implant, temporary pacing |
| Phrenic nerve palsy | phrenic, diaphragm palsy, diaphragm paralysis, diaphragm elevation |
| Vascular access complications | hematoma, pseudoaneurysm, retroperitoneal bleed, arteriovenous fistula, access site bleed, femoral hematoma |
| Atrial flutter/arrhythmia recurrence | atrial flutter, flutter, arrhythmia recurrence, AF recurrence, recurrent atrial fibrillation |
| Esophageal/atrioesophageal injury | esophageal, oesophageal, atrioesophageal, esophageal fistula |
| Pulmonary vein stenosis | pulmonary vein stenosis, PV stenosis, pulmonary vein narrowing |

**Supplementary Table 2.** Device Malfunction Subtypes: Classification Criteria and Report Distribution

| **Malfunction Subtype** | **Pooled, n (%)** | **PulseSelect, n (%)** | **Farapulse, n (%)** | **Sphere-9/Affera, n (%)** | **Varipulse, n (%)** |
| --- | --- | --- | --- | --- | --- |
| Retraction/retrieval failure | 802 (38.8) | 114 (19.6) | 657 (48.9) | 11 (20.8) | 20 (22.7) |
| Electrical/energy delivery failure | 209 (10.1) | 171 (29.4) | 13 (1.0) | 6 (11.3) | 18 (20.5) |
| Mechanical fracture/damage | 275 (13.3) | 78 (13.4) | 184 (13.7) | 4 (7.5) | 9 (10.2) |
| Connector/interface failure | 87 (4.2) | 52 (9.0) | 34 (2.5) | 0 (0.0) | 1 (1.1) |
| Deployment/delivery failure | 120 (5.8) | 10 (1.7) | 107 (8.0) | 1 (1.9) | 2 (2.3) |
| Contamination/sterility breach | 51 (2.5) | 4 (0.7) | 44 (3.3) | 3 (5.7) | 0 (0.0) |
| Thrombus/clot formation | 20 (1.0) | 0 (0.0) | 12 (0.9) | 2 (3.8) | 6 (6.8) |
| Fluid/perfusate/irrigation failure | 16 (0.8) | 1 (0.2) | 14 (1.0) | 0 (0.0) | 1 (1.1) |
| Software/sensor failure | 17 (0.8) | 1 (0.2) | 5 (0.4) | 7 (13.2) | 3 (3.4) |
| Catheter tip/electrode issue | 13 (0.6) | 6 (1.0) | 4 (0.3) | 2 (3.8) | 1 (1.1) |
| Other/unspecified | 458 (22.1) | 144 (24.8) | 269 (20.0) | 17 (32.1) | 27 (30.7) |
| **Total malfunction reports** | **2,068** | **581** | **1,343** | **53** | **88** |
| *Denominator for pooled percentages is total malfunction reports (n = 2,068). Device-level percentages reflect the proportion of that device's malfunction reports assigned each subtype. Subtypes are assigned by first-match keyword classification of the concatenated MAUDE narrative; each report receives exactly one subtype. The Other/Unspecified category includes reports with no keyword match, absent narrative, or patient complication events without an identifiable device hardware failure mode. The Sphere-9/Affera system supports radiofrequency in addition to pulsed field energy; its subtype distribution reflects RF-specific failure modes (steam pop, thermal events) not represented in the primary taxonomy.* | | | | | |
